# Nurses’ pain management practices for admitted patients at the Comprehensive specialized hospitals and its associated factors, a multi-center study

**DOI:** 10.1186/s12912-023-01528-x

**Published:** 2023-10-06

**Authors:** Legese Fekede, Worku Animaw Temesgen, Haileyesus Gedamu, Selamsew Kindie, Tola Getachew Bekele, Ambaw Abebaw, Aemiro Baymot, Mesfin Difer

**Affiliations:** 1 Department of Nursing, College of Medicine and Health Sciences, Wolkite University, Wolkite, Ethiopia; 2 Department of Nursing, College of Medicine and Health Sciences, Bahir Dar University, Bahir Dar, Ethiopia; 3 Department of Nursing, College of Medicine and Health Sciences, Mada Wulabu University, Bale Robe, Ethiopia; 4 Department of Nursing, Jimma University Medical Center, Jimma, Ethiopia; 5 Department of Nursing, College of Medicine and Health Sciences, Addia Ababa University, Addis Ababa, Ethiopia

**Keywords:** Pain, Hospitalized patients, Nurses, Pain management practice, Amhara region

## Abstract

**Background:**

Pain is the most common challenge that most hospitalized patients complain of and is influenced by several patients, nurses, and institutional-related factors. Most studies in Ethiopia on pain were focused on surgical illnesses only.

**Objective:**

To assess nurses’ pain management practice and associated factors for admitted patients at Comprehensive Specialized Hospitals.

**Methods and materials:**

: A multi-center institution-based cross-sectional study was conducted at the five randomly selected Comprehensive Specialized Hospitals of the Amhara region from May 01 to June 01, 2022. A multi-stage sampling method was employed to select a total of 430 nurses and patients for whom the nurses were responsible. Data were collected using standard self-administered, structured, and checklist questionnaires from nurses, patients, and patients’ charts respectively. The modified Bloom’s criteria categorized the overall practice as good, moderate, and poor. Data were checked, coded, and entered into Epi-Data version 4.6 and exported to SPSS version 25. An ordinal logistic regression model was applied, and variables with a p-value < 0.05 with a 95% CI in the multivariable analysis were considered significant.

**Results:**

The study evaluated the pain management practices of 430 nurses and only a quarter had good pain management practices. Those nurses with first degrees and above education level (AOR = 2.282) and who attended in-service training (AOR = 2.465) were found to have significantly higher pain management practice. Expected though patients with painful procedures (AOR = 5.648) and who had severe pain (AOR = 2.573) were receiving better pain management practices from their nurse care provider. Nurses working in the institutions with a pain-free initiative focal person (AOR = 6.339) had higher pain management practices.

**Conclusion and recommendation:**

: Overall, the majority of nurses had poor pain management practices. Higher educational levels, in-service training, and assigning a pain-free focal person had an impact on pain management services. Patients with higher pain levels and painful procedures were getting better attention. Hospital administrations need to provide due attention to the pain management of hospitalized patients by providing in-service training and educational opportunities to improve the capacity of nurses. Patients would be benefited considerably if hospitals focus on assigning focal persons for advocating regular pain management for admitted patients regardless of their pain level.

**Supplementary Information:**

The online version contains supplementary material available at 10.1186/s12912-023-01528-x.

## Introduction

The International Association for the Study of Pain (IASP) defined pain as an unpleasant sensory and emotional experience associated with actual or potential tissue damage or described in terms of such damage [1]. It is a subjective sensation caused by high-intensity stimuli triggering pain receptors in the skin, muscle, bone, and other tissues [2]. Pain is now recognized as the “fifth vital sign,“ which is assessed and managed regularly. If it is not treated timely and properly, pain affects almost every aspect of the life of the patient as well as the family [3, 4].

Pain management practices refer to those activities done by health care providers, particularly nurses to relieve those who are in pain, which includes pain assessment using organizationally accepted tools, providing pharmacological and non-pharmacological pain management, and evaluation of pain management by reassessing patients’ status. It is effective when patient outcomes, satisfaction levels, and quality of life are all improved [5, 6].

Despite regular pain assessments and management protocols, the burden of pain among hospitalized patients remains significant. More than half of admitted patients complain of pain during most of their hospital stay (7–10). Certain studies in different areas including Ethiopia revealed that nurses’ pain management practice for hospitalized patients is inadequate [11–14].

There have been different patient-related factors influencing the pain management practices of nurses according to the findings of different studies in Ethiopia. For instance, patients’ gender was one of the factors in which being female was about five times higher level of pain as compared to males which in turn influenced the pain management practices of nurses according to Debre Tabor of Ethiopia (15). A study conducted in Gondar revealed that pain severity had a significant association with the nurses’ pain management practices [16].

According to the findings of different studies; knowledge of nurses to the pain assessment and management, age, educational qualification, work experience, and in-service training were nurse-related factors that had significant association with the pain management practices of nurses for hospitalized patients [11, 13, 17–20]. Nurse-to-patient ratio was an institutional-related factor which was indicated by insufficient number of patients for the number of patients was associated with inadequate pain management practices [13, 20, 21].

The factors; particularly the patient-related factors that influence the nurses’ pain management practices were not studied well in Ethiopia. Another limitation of Ethiopian studies on pain management practices is they frequently rely on surgical patients, particularly post-operative ones. Therefore, the current study aimed to investigate pain management practices and factors that act as barriers or facilitators to nurses’ pain management practices which subsequently increase the burden of pain among hospital admitted patients.

## Methods and materials

### Study design and setting

An institutional-based multi-center cross-sectional study was conducted among nurses working at five randomly selected referral hospitals (Tibebe-Ghion, Felege-Hiwot, Debre-Tabor, Debre-Markos, and Debre-Berhan) of Ethiopia and patients admitted at these hospitals from May 01 to June 01, 2022.

### Inclusion criteria

All nurses working in the inpatient departments of the selected Hospitals during the study period were considered in this study.

All patients admitted to the inpatient departments of the selected Hospitals during the study period were considered in this study.

### Exclusion criteria

Nurses on annual leave or sick leave during the data collection period were excluded from the study. Patients with critical illness and impaired level of consciousness were excluded from the study.

### Sample size and sampling

The sample size was determined using a single population proportion formula by taking a proportion of 23.53% from Jimma University Medical Center (JUMC) [11]. By adding a 5% non-response rate to the initial sample, the sample size becomes 291. The sample was again multiplied by a design effect of 1.5 yielding the final sample size of 437, which is the number of nurses who were included in the study.

The same number (437) of patients were included in the sample to assess pain intensity directly from the patients and the pain management practices of nurses from the corresponding patient’s chart.

Nurses play a key role in the management of patient’s pain and they are in charge of patient care during their scheduled working hours as well as the patients that they are assigned for. The pain management practice of nurses was evaluated from the records of the patient’s charts for whom he/she was responsible. That is why an equal number of nurses and patients were intended to be studied. Before selecting a nurse for the study, the bed numbers that he/she was responsible for were determined. Then, one of the patients for whom the nurse is responsible was chosen at random so that the chart could be reviewed and patient-related characteristics could be evaluated. From the patients’ charts, data about administered medications such as anti-pains and vital signs incorporating the latest pain score were collected. The charts were reviewed about four hours back to evaluate the corresponding nurses’ pain management practices. During this time the nurse involved in this study was assumed to be responsible for the care and records that have been done to the patients participated. Because nurses are only assigned to a certain number of patients for a specified amount of time in hospital settings.

### Sampling procedure

A multi-stage sampling technique was employed to select the study samples. First of all, a list of all comprehensive specialized hospitals had been obtained from the Amhara health bureau. Five hospitals (Tibebe-Ghion, Felege-Hiwot, Debre-Tabor, Debre-Markos, and Debre-Berhan) were selected randomly from the eight comprehensive specialized hospitals. Following that, each institution provided the number of nurses employed in both the outpatient and inpatient departments who rotated between the two settings. Then the calculated sample size was proportionally allocated to the size of the population in the selected hospitals. Five inpatient departments (Medical, Surgical, orthopedics, Oncology, and Maternity) were selected in each hospital, and nurses working in those departments again were allocated proportionally based on their number. Finally, simple random sampling by lottery method was employed to select the study samples.

**Fig. 1:**
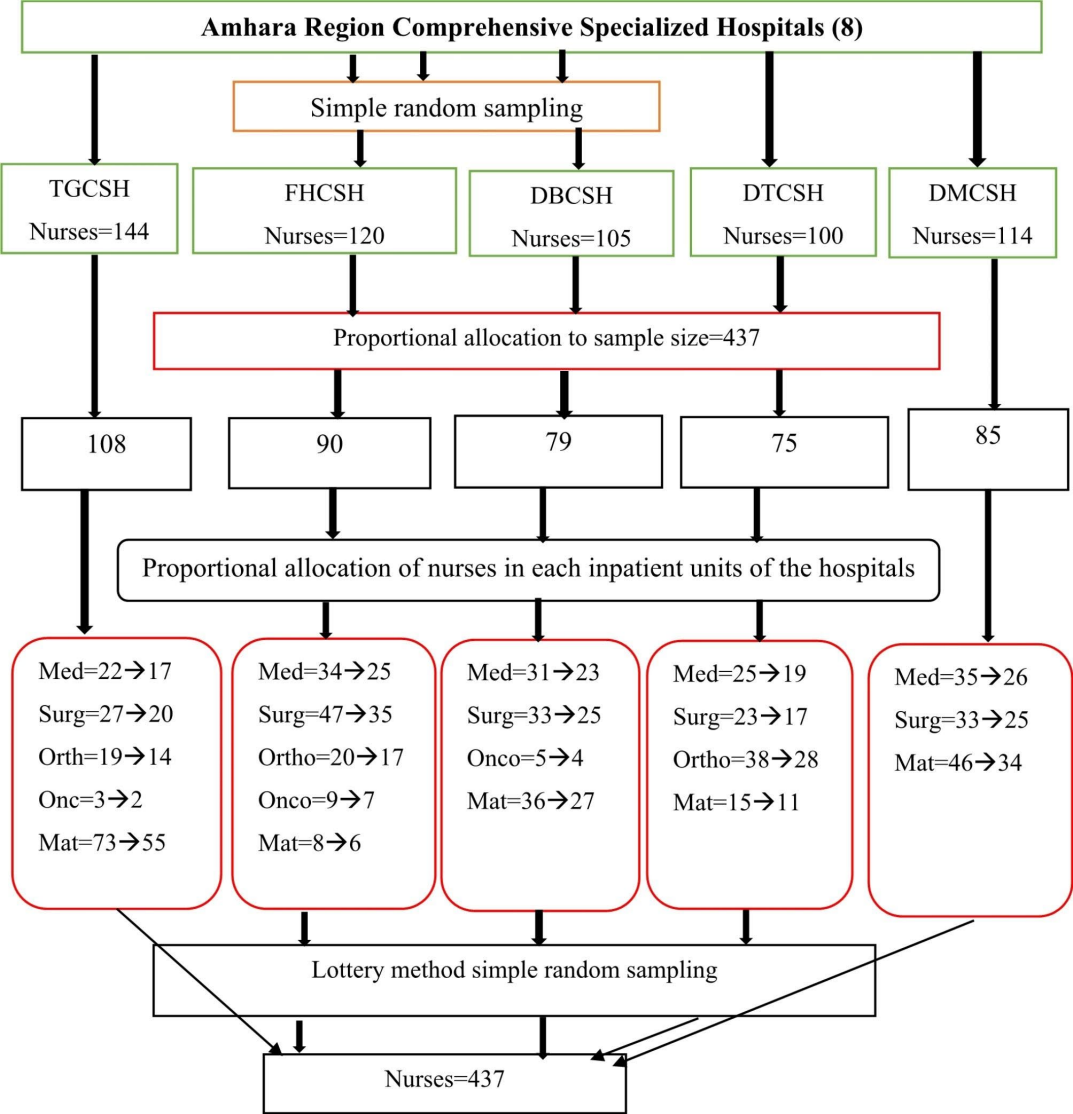
Schematic presentation of sampling procedure on pain management practices and associated factors of nurses for admitted patients at the Comprehensive Specialized Hospitals of the Amhara region of Ethiopia, 2022

The total number of patients admitted per month was obtained from each department’s Health Management Information System (HMIS) database. A total of 437 patients with their hospital patient charts were selected from the selected units of each hospital to determine pain intensity. Before selecting a nurse for the study, the bed numbers he/she was responsible for were determined. Then, one of the patients that the nurse is responsible for was selected. The details of the sampling procedure is presented below Figure 1.

### Study variables

#### Dependent variable

Nurses’ pain management practice.

### Independent variables

Independent variables are categorized into three groups, which include:-

#### Patient-related variables

Socio-demographic variables (age, sex, educational status, marital status, religion, occupation) and Clinical factors (painful procedure, latest pain score record, type of analgesics, and pain intensity).

#### Nurse-related variables

Socio-demographic characteristics (age, sex, educational status, work experience, and working unit), and knowledge were nurses-related variables.

#### Institutional-related variables

In-service training, nurse-to-patient ratio, written policy for pain as the “5th vital sign”, pain assessment tools, pain management guidelines, number of working shifts, and pain-free hospital initiative implementation focal person were institutional-related variables.

### Operational definitions

#### Patients with severe pain

are those patients who are in pain whose pain score ranged from seven to ten [7–10] numerically [22, 23].

#### Good pain management practice

According to the checklist evaluation, nurses with scores ≥ 75% were regarded as having good pain management practice [12, 24, 25].

#### Moderate pain management practices

Based on the checklist evaluation, nurses with scores between 50 and 74.99% were considered as having moderate pain management practices [12, 25].

#### Poor pain management practice

Based on the checklist evaluation, nurses with scores less than 50% were considered as having poor pain management practices [12, 24, 25].

#### Adequate nurse to patient ratio

When a nurse serves ≤ 6 patients in all units other than the Intensive Care Unit (ICU) which is based on Ethiopian Standard Agency (ESA) for Comprehensive hospitals [26, 27].

#### Good knowledge

Those nurses who scored above or equal to 80% of the knowledge questions were regarded as having adequate knowledge about pain management [24].

#### Painful procedure

Any procedure that could reasonably be anticipated to result in more than mild or transient pain and/or distress in the patients to whom it is applied [28].

### Data collection tools and procedures

Data were collected using a self-administered questionnaire from nurses and an interviewer-administered questionnaire from patients as well as a checklist for chart review and observation. Some parts of the questionnaire prepared for this study whereas, the other parts were taken from previous studies. An 8-item binary scoring (yes/no) data extraction checklist was used to evaluate nurses’ pain management practices. Those who scored ≥ 75% [6] were regarded as a good pain management practice, those who had a score of 50-74.9% (4–5) were regarded as moderate practice, and those who had a score of < 50% [4] poor pain management practices. The tool was adapted from the Pain-Free Initiative Implementation Manual and different studies in Ethiopia [12, 24]. A 12-item with binary scoring (yes/no) questionnaire was used to assess the level of nurses’ knowledge on pain management. Those nurses who scored ≥ 80% (10) of the knowledge questions were regarded as having good knowledge. It was adapted from different Ethiopian studies (12, 24). The WHO pain ladder was used as a tool to assess the pain intensity of patients and was reported as no pain (zero), mild pain (one-three/1–3), moderate pain (four-six/4–6), and severe pain (seven-ten/7–10) (23, 29). A 6-item checklist was used to determine institutional-related factors to nurses’ pain management practices [11, 13, 30]. The questionnaire also contains the socio-demographic characteristics of nurses and the sociodemographic and clinical characteristics of patients. Four who were not working at study hospitals collected the data after a full day training.

### Data quality control

The data quality was maintained by using a carefully designed questionnaire collected by well-trained data collectors and supervisors. Three diploma nurses and two BSC nurses were recruited to collect and supervise the data collection process in each hospital. The data collectors and supervisors were trained for one day on procedures, techniques, and ways of collecting the data. Data were checked for completeness and consistency. A pretest was conducted at the University of Gondar Comprehensive Specialized Hospital (UoGCSH) on 22 nurses.

### Data analysis and processing

Data were checked, coded, and entered into Epi-Data version 4.6 and exported to SPSS version 25 for analysis. Descriptive statistics such as frequency distribution, proportion, and percentages were done. The extent of multi-collinearity between independent variables was checked using variance inflation factor (VIF) and tolerance and the values of both statistics for all independent variables were within an acceptable range (VIF < 10 and Tolerance > 0.1). Regression model assumptions were checked using a test like the Hosmer & Lemeshow goodness of fit with a P-value (Pearson chi-square = 0.096 and Deviance-0.776). The proportional odds assumption was checked with a test of parallel lines with a P-value ≥ 0.05 which is an indicator for the fulfillment of the assumption. A P-value for the test of parallel line for this study was 0.421.

A bivariable ordinal logistic regression model was fitted for each explanatory variable. Accordingly, those variables having a p-value ≤ 0.25 in the bivariable regression analysis were taken as the candidates for the multivariable ordinal logistic regression model. In multivariable regression, variables with a P-value of less than 0.05 with a 95% CI were considered statistically significant.

## Result

### Socio-demographic characteristics of nurses and patients

The study included 430 nurses in total, with a response rate of 98.4%. Over half 228 (53%) were males and the mean age was 30.44 with a standard deviation (SD) of 4.311. Majority 374 (87%) of nurses had an educational qualification of degree and above. Majority 206 (47.9%) of nurses had a work experience of 5–10 years (mean = 7.59, SD = 4.05). Only 58 (13.5%) of nurses had attended in-service training regarding pain management in the past two years (Table 1).

In this study, 430 patients were interviewed, their pain level was assessed using NRS, and the charts of the corresponding patients were reviewed. The majority were females 260 (60.5%) and the mean age was 45.32 years with a range of 18–86 years. Regarding the educational status of the patients, 148 (34.4) were unable to read and write, while 132 (30.7%), 67 (15.6%), and 83 (19.3%) had primary, secondary, and diploma and above educational levels respectively. Most of the patients were farmers (25.3%) and housewives (35.1%) (Table 1).

**Table 1:** Socio-demographic characteristics of nurses (n = 430) and patients (n = 430) at Amhara Region Comprehensive Specialized Hospitals 2022

Nurses’ socio-demographic characteristics					Patients’ socio-demographic characteristics			
Variables	**Category**	**n**		**%**	**Variables**	**Category**	**n**	**%**
Gender	Male	228		53.0	Gender	Male	170	39.5
	Female	202		47.0		Female	260	60.5
Age	21–30	274		63.7	Age	18–35	177	41.2
						35–555	67	15.6
	≥ 31	156		36.3		≥ 56	186	43.3
Education	Diploma	56		13.0	Education	Unable to read & write	148	34.4
	Degree & above	374	87.0			Primary education	132	30.7
						Secondary education	67	15.6
						College & above	83	19.3
Experience	0–4 years	150		34.9	Marital status	Married	323	75.1
	5–10 years	206		47.7		Single	65	15.1
						Divorced	25	5.80
	≥ 11 years	74		17.2		Widowed	17	4.0
Working unit	Medical	113		26.3	Occupation	Gov’t employee	102	23.7
	Surgical	122		28.4		Housewife	140	32.6
	Orthopedics	58		13.5		Farmer	127	29.5
	Oncology	19		4.4				
	Maternity	118		27.4		Merchant	45	10.5
In-service training	Yes	58		13.5		Others	16	3.7
	No	372		86.5				

### Knowledge of nurses toward pain management practices

The mean nurses’ knowledge of pain management score was 8.84 with a standard deviation of 1.92. The most correctly answered item was “Pain should be assessed before and after administering anti-pain drugs” with a 98.8% correct response rate. The question “Acetaminophen and non-steroidal anti-inflammatory agents are effective analgesics for severe pain” was the least answered item with only a 24% correct response rate. Of all nursing professionals, the majority 273 (63.5%) had poor knowledge of pain management practices ((Table 2).

**Table 2:** Knowledge of nurses toward pain management practices for admitted patients (n = 430) at Amhara Region Comprehensive Specialized Hospitals, 2022 G.C.

Variables	Category	n	%
Patients are the most accurate judge of their pain	Yes	246	57.2
Currently, pain is regarded as one of the vital signs	Yes	421	97.9
Pain assessment before and after administering anti-pain	Yes	425	98.8
Placebo is helpful to assess a patient if he/she is really in pain	Yes	122	28.4
The patient should be advised to use non-drug techniques with pain medication	Yes	398	92.6
Distraction using relaxation can decrease the perception of pain	Yes	384	89.3
Side effects of opioids should be observed at least 20–30 min after administration	Yes	354	82.3
Combining analgesics may result in better pain control with fewer side effects	Yes	198	46.0
providing comfort and positioning may help to reduce pain	Yes	419	97.4
Assessment is the priority for effective pain management	Yes	400	93.0
Acetaminophen and NSAIDs are not effective for severe pain	Yes	103	24.0
Subsequent doses of anti-pain should be adjusted according to the patient’s response	Yes	360	83.7
Level of nurses’ knowledge of pain assessment and management	Good	157	36.5
	Poor	273	63.5

### Clinical characteristics of patients

Regarding the clinical characteristics of patients, only 207 (48.1%) of all patients’ pain was recorded within 24 h before the chart review. More than half of 238 (55.3%) patients had undergone painful procedures. When assessed using NRS, 351 (81.63%) had reported mild to severe pain with a median and IQR of 3 and 4 respectively ((Table 3).

**Table 3:** Clinical characteristics of patients to nurses’ pain management practices and associated factors for admitted patients (n = 430) at Amhara Region Comprehensive Specialized Hospitals, 2022

Variables	Category	Frequency	Percent
Latest pain record	Yes	207	48.1
	No	223	51.9
Type of anti-pain	Non-opioids	76	17.7
	Weak opioids	154	35.8
	Strong opioids	30	7.0
	Mixed	42	9.8
	None	128	29.8
Patients with a painful procedure	Yes	192	44.7
	No	238	55.3
Pain intensity	No pain	79	18.4
	Mild pain	163	37.9
	Moderate pain	106	24.7
	Severe pain	82	19.1

### Institutional-related factors for pain management practices

Five of the Comprehensive Specialized Hospitals of the Amhara region were included in the study. 4 (80%) of the five hospitals had two working shifts and 3(60%) hospitals had a 1 to 6 nurse-to-patient ratio for working hours. Of those hospitals, 4 (80%) had a written policy for pain as the 5th vital sign, 3 (60%) had pain management guidelines, and only one (20%) of the institutions had a pain-free hospital initiative focal person.

### Pain management practices of nurses

About 222 (51.6%) of the charts revealed pain was assessed and recorded at least once within the past four hours by the nurses. Among those nurses evaluated by chart review of their particular client, only 152 (35.3%) had performed a regular pain assessment during their stay at the workplace. Regarding the level of practice, only 111 (25.8%) of nurses had good pain management practices while 118 (27.4%) and 201 (46.7%) had moderate and poor pain management practices respectively ((Table 4).

**Table 4:** Pain management practices nurses for admitted patients (n = 430) at Amhara Region Comprehensive Specialized Hospitals, 2022

Variable	Category	Freq	%
Pain score sheet is attached and incorporated with the patient’s chart	Yes	427	99.3
Pain assessment and recording using the standard pain assessment tools at least once over the past 4 h	Yes	222	51.6
Regular pain assessment for the particular patient	Yes	152	35.3
A patient with pain provided anti-pain medications	Yes	292	67.9
Pain managed based on WHO pain management standards	Yes	125	29.1
Non-pharmacological pain management	Yes	124	28.8
Pain management before and after every procedure	Yes	120	27.9
Monitoring side effects of anti-pain medications as per protocol	Yes	7	1.60

### Factors associated with Nurses’ pain management practice

A total of 23 variables were included in the model of bivariate ordinal logistic regression level. On the multi-variable ordinal logistic regression, five (5) variables become significantly associated with pain management practices at a P-value less than 0.05. These variables are nurses’ educational level, in-service training, patients with the painful procedure, pain intensity, and assigned pain-free initiative implementation focal person in the institutions.

The educational status of nurses was found to have a statistically significant relationship with the pain management practices of nurses. The odds of being in a higher level of pain management practice was 2.282 times (AOR-2.282, with 95%CI: [1.181–4.41]) higher on average for those having an educational qualification of degree and above as compared to those having a diploma.

In-service pain management training was found to be significantly associated with pain management practices. The odds of being in a higher category of pain management practices was 2.47 times (AOR = 2.465 with 95%CI:[1.317–4.614]) higher on average for those nurses who attended an in-service pain management training as compared to those who didn’t attend. In other words, those nurses who attended pain management training had a 71% probability of having a higher level of pain management practices.

From the patient-related factors, patients with painful procedures become a significant predictor of nurses’ pain management practices. The odds of being in a higher category of pain management practice was 5.648 times (5.648 with 95%CI: [3.237–9.856]) higher for those nurses who provide care for patients with painful procedures as compared to the contrary group. Those nurses who follow those patients with painful procedures had an 85% probability of falling in the higher category of pain management practices.

The other patient-related significant variable was pain intensity. The odds of being in a higher level of pain management practice was 2.573 times (2.573 with 95%CI: [1.35-5-4.899]) higher on average for those nurses who follow patients with severe pain as compared to those who follow patients with mild pain. Similarly, the odds of being in a higher category of pain management practice was 3.236 times (3.236 with 95%CI: [1.771–5.914]) higher on average for those nurses who follow patients without pain as compared to those who follow patients with mild pain.

From institutional factors, a pain-free initiative implementation focal person was found to have a statistically significant relationship with the nurses’ pain management practices. The odds of being in a higher category of pain management practice was 6.34 times (6.339 with 95%CI: [3.611–11.13]) higher on average for those nurses working in the institutions having a pain-free hospital initiative implementation focal person as compared to the contrary group. Those nurses working in the hospitals having a pain-free hospital initiative implementation focal person had an 86.4% probability of having a higher level of pain management practices (Table 5).

**Table 5:** Bivariable and Multi-variable ordinal logistic regression analysis to factors associated with pain management practices of nurses for admitted patients (n = 430) at Amhara Region Comprehensive Specialized Hospitals, 2022

Variables	Category	Pain mgt			COR (95%CI)	AOR (95%CI)	P-value
		1	2	3			
Nurses’ Education	Diploma	36	10	10	1		
	≥Degree	165	108	101	2.136[1.207–3.782]*	2.282[1.181–4.41]**	0.014
Working experience	0–10 years	172	91	82	0.557[0.360–0.862]*	0.76[0.451–1.282]	0.304
	≥ 11 years	29	27	29	1	1	
Working unit	Medical	62	28	23	1.791[1.066–3.007]*	2.163[0.995-4.70]	0.051
	Surgical	39	33	50	4.975[2.99–8.28]*	1.667[0.794-3.50]	0.177
	Ortho	15	22	21	5.035[2.756–9.199]*	2.136[0.946–4.82]	0.068
	Oncology	6	7	6	4.042[1.649–9.905]*	1.96[0.629–6.11]	0.246
	Maternity	79	28	11	1	1	
In-service training	Yes	14	18	26	2.910[1.736–4.877]*	2.465[1.317–4.614]**	0.005
	No	187	100	85	1	1	
Knowledge	Good	52	51	54	2.234[1.545–3.229]*	1.305[0.826–2.061]	0.254
	Poor	149	67	57	1	1	
Age of patients	18–35	96	44	37	0.545[0.369–0.805]*	0.997 [0.587–1.692]	0.991
	36–55	32	21	14	0.652[0.389–1.095]	0.671[0.367–1.228]	0.196
	≥ 56	73	53	60	1	1	
Patient gender	Male	67	45	58	1.775[1.234–2.553]*	1.282 [0.795–2.065]	0.308
	Female	134	73	53	1	1	
Painful procedure	Yes	45	65	82	5.887[3.994–8.677]*	5.648[3.237–9.856]**	0.001
	No	156	53	29	1	1	
Pain intensity	No pain	28	22	29	4.143[2.434–7.052]*	3.236[1.771–5.914]**	0.001
	Severe	12	28	42	9.130[5.337–15.62]*	2.573[1.35–5.4.899]**	0.004
	Moderate	51	36	19	2.045[1.264–3.307]*	1.022[0.574–1.82)]	0.942
	Mild	110	32	21	1	1	
Pain mgt guide	Yes (277)	118	79	79	1.554[1.069–2.258]*	1.471[0.901-2.40]	0.123
	No (153)	83	39	32	1	1	
Focal person	Yes (317)	16	30	44	4.41[2.884–6.934]*	6.339[3.611–11.13]**	0.001
	No	185	88	67	1	1	

Key note: 1-poor, 2-moderate, 3-good

## Discussion

This study aimed determine nurses’ pain management practices and associated factors for admitted patients at the Amhara region comprehensive specialized hospitals. The study indicated that only 111 (25.8%) with a 95%CI [0.217, 0.302]) of the nurses had good pain management practices, while 118 (27.4%) and 201 (46.7%) had moderate and poor pain management practices respectively. The finding of this study was consistent with the study conducted at Hawassa Referral Hospital (24.4%) [12]. However; it was lower compared to the studies done at the Federal Hospitals of Addis Ababa (56.5%), governmental hospitals of Harari region and Dire Dawa city administration (33.6%), and Jimma University Medical Center (JUMC) (76.47%) [11, 13, 31].

This gap could be explained by the difference in sample size, as the sample sizes in the earlier research were generally less than those in this study. On the other hand, the discrepancy may have arisen from the use of an objective tool to evaluate nurses’ pain management practices in the current study. The disparity in the study populations may be another factor contributing to the discrepancy. The studies done in Addis Ababa (13) was on the practices of nurses for critically ill patients and that of Jimma (11) and Dire Dawa (31) were for post-operative patients. Whereas the current study was in all adult inpatient units as well as multicenter. Patients with critical illnesses typically need to be closely monitored on a regular basis, which forces nurses to pay attention to their patients’ pain levels and treat them appropriately. On the other hand, compared to patients without critical illness, post-operative patients, critically sick patients, and those with more severe pain tend to report their pain status and could be managed. However, pain management might not be as effective as it could be in the case of patients who are unable to report and have critical illnesses [16, 32, 33].

This study was multicenter which was in contrast to the studies of Jimma, Hawassa, and Dire Dawa which might result in a difference in the level of nurses’ pain management practices. The other reason that might have brought the difference in the level of nurses’ pain management practices could be the difference in the provision of pain management training. For example, only 13.5% of professionals attended pain management training in this study which was lower compared to the finding of Jimma in which 23.5% of professionals had attended in-service pain management training which might have resulted in the difference in the level of pain management practices of nurses.

The result of this study was relatively higher as compared with the study done in Rwanda in which only 2% of the study participants had good pain management practices (14). The possible reason for the discrepancy could probably be attributed to the educational level of nurse professionals in Rwanda in which only 4% of nurse professionals had an educational qualification of degree which is far less than the current study in which 87% had a degree and above level of education. When patients’ pain is not managed sufficiently, they may experience serious negative effects, including socioeconomic, clinical, and emotional ones. It could lead to chronic persistent pain, unexpected readmission, and delayed recovery. Regular assessment and management of pain are essential for a better patient outcome [34].

As expected the educational status of nurses was found to have a statistically significant relationship with the nurses’ pain management practices. The odds of being in a higher level of pain management practice was nearly 2.3 times higher on average for those with educational qualifications of a degree and above than those with a diploma. The possible reason would be that nurses with a degree and higher levels of education typically have better knowledge and comprehension of pain, its assessment, and management mechanisms, which in turn leads to better pain management practice. A higher level of education has an impact on nurses’ pain management practices, i.e. if someone is not equipped with the necessary knowledge and skills important to manage pain, patients will not get an appropriate pain management service from him/her and end up suffering from pain. The knowledge and clinical skills of professionals will be greatly enhanced by advanced nursing education, which will lead to better pain management for patients. Patients would also benefit when they get attention to their pain from skillful professionals throughout their hospital stay. The finding was supported by the studies conducted in Hawassa and Rwanda [12, 14].

This study indicated that in-service pain management training was found to be significantly associated with pain management practices. A higher category of pain management practice was more likely for nurses who participated in in-service pain management training compared to those who did not in the previous two years, by a factor of 2.47. This is because in-service training can provide nurses with more knowledge, insight, and understanding of patients’ pain, which may enhance their pain management practices. In other words, it serves to update the nurses’ pain management knowledge and practical skills and improve the best practices for fulfilling their responsibilities in the regular assessment and management of pain for patients. It also plays an essential role in improving the standard of inpatient pain management practices [35, 36]. This finding was supported by the study conducted at Jimma University, the Federal Hospitals of Addis Ababa, and the two public hospitals in the Oromia region [11, 13, 37].

In this study, patients with painful procedures become a significant predictor of nurses’ pain management practices. Nurses who care for patients with painful procedures had 5.65 times higher odds of falling into a higher category of pain management practice than the opposite group. This could probably be because painful procedures increase the likelihood of high-intensity of pain that drives patients to report their pain to their care providers. Nurses who take part in caring for patients with painful procedures might be able to reduce the suffering of patients with procedural pain which put them in the higher category of pain management practices [38].

The other patient-related significant variable was pain intensity. Nurses who care for patients with severe pain had, nearly 2.6 times higher odds of practicing higher levels of pain management than nurses who care for patients with mild pain. The possible reason might be that patients with severe pain are more likely to report their pain status and get more attention from their healthcare providers. According to findings of studies in Gondar [16] and Central Africa [33], patients with severe pain are managed more frequently, relatively early, and regularly than those with mild and moderate ones which puts nurses who follow patients with severe pain in the higher category of pain management practices.

From institutional factors, a pain-free initiative implementation focal person was found to have a statistically significant impact with the nurses’ pain management practices. The odds of being in a higher category of pain management practice was nearly 6.34 times higher for those nurses working in the institutions having a pain-free hospital initiative implementation focal person as compared to the contrary group. The possible reason behind this could probably be that nurses may improve their pain management activities if they are guided by trained professionals who feel responsible for the proposed area of practice. Additionally, the pain-free hospital initiative would be implemented when there is a responsible individual who can advocate a regular patient assessment and management of patients with any pain level. Those who are assigned as focal persons have vital importance in the improvement and continual provision of pain management for hospitalized patients. This could be achieved by preparing a work plan, evaluating pain management activities, and coordinating patient education programs [36, 39].

## Conclusions and recommendation

This study found that the majority of nurses had poor pain management practices for admitted patients. Nurses with higher education level and having in-service training were providing better pain management to their admitted patients. Patients with painful procedures and higher pain intensity were receiving better pain management from their nurse care provider. From several institutional factors, the assigned pain-free hospital initiatives implementation focal person was another factor that remained significant to the pain management practices of nurses.

Hospitals administrations need to provide due attention to the pain management of hospitalized patients by providing in-service continuous training to improve the capacity of the health care providers. It would improve the service of pain management for admitted patients if there is a focal person for a pain-free hospital initiative implementation who can advocate a regular pain assessment and management for each admitted patient regardless of their pain severity. Finally, future researchers would get better findings if they perform an observational follow-up study to determine the pain management practices of nurses.

### Limitations of the study

The study used only patients’ charts to evaluate nurses’ pain management practices for their corresponding patients as it was difficult to observe and follow all of the nurses within the specified period. Social desirability bias during data collection was another drawback of this study. The other limitation was that it didn’t include the pain management practices for patients who were with critical illness and impaired level of consciousness since the pain assessment tool (NRS) was not suitable for them.

### Electronic supplementary material

Below is the link to the electronic supplementary material.

Supplementary Material 1

## Data Availability

The datasets created and analyzed during the current study are available from the corresponding author upon a reasonable request.

## Abbreviations

AOR: Adjusted Odds Ratio
CI: Confidence Interval
COR: Crude Odds Ratio
IASP: International Association for the Study of Pain
NRS: Numerical Rating Scale
SD: Standard Deviation
SPSS: Statistical Package for the Social Sciences
UoGCSH: University of Gondar Comprehensive Specialized Hospital
VIF: Variance Inflation Factor
WHO: World Health Organization
