# Supplementary material for: Nurses’ pain management practices for admitted patients at the Comprehensive specialized hospitals and its associated factors, a multi-center study
Source: BMC Nurs. 2023 Oct 6;22:366. doi: 10.1186/s12912-023-01528-x (PMC10559436; doi:10.1186/s12912-023-01528-x)
Supplement: Supplementary file 1 — Supplementary Material 1 [file 12912_2023_1528_MOESM1_ESM.docx]

# **Appendix: Questionnaire**

**Appendix A: participant information sheet and consent form**

Bahir Dar University, college of Medicine and Health Sciences, Department of Adult Health Nursing

Name of the Principal Investigator: Legese Fekede Abza

Name of the organization: Bahir Dar University, college of medicine and health science, department of Adult Health Nursing.

**Information Sheet prepared for participants in the Comprehensive specialized hospitals of Amhara region**

My name is ______. I am here representing Legese Fekede, Adult Health Nursing Student at Bahir Dar University, College of Health Sciences, department of adult health nursing, Graduate Study Program, and he is conducting a research on a topic entitled as “Nurses’ Pain Management Practices for Admitted Patients at the Comprehensive Specialized Hospitals and its Associated Factors”.

I am inviting you to participate in this research. You may choose to take part or not and if you choose to, you are free to withdraw from the study at any time during the study. If you do not want to take part, your service in the hospital will not be affected by your decision.

The necessary information regarding the study is mentioned below.

**Purpose of the study:** the purpose of this study is to assess pain management practices and associated factors of nurses for admitted patients at the Comprehensive Specialized Hospitals of Amhara Region.

**Benefits and risks of the study:**

**Benefits:** For your participation in the study no payment will be granted. Your responses to the following questions are beneficial to you, other patients, as well as to a good nursing outcome.

**Risks:** Your participation will not cause any harm to you. The study will be conducted by a self-administered questionnaire and it may take 15 – 25 minutes to complete the forms.

**Confidentiality**: to establish the confidentiality of research data, the principal investigator (PI) will use codes during the data collection period instead of using names. So the information you provide for us will be kept strictly confidential.

**Right of the participant**: Participating and not participating in the study is the full right and participants can stop from participating in the study at any time. This would not affect at all your health and nobody will enforce you to explain the reason for withdrawal.

**Person to contact:** This research work is approved by the institutional review board of the college of medicine, and health sciences of Bahir Dar University. If you have any questions you can contact any of the following individuals (Investigator and Advisors) and you may ask at any time you want.

Legese Fekede: Adult Health Nursing student at Bahir Dar University

Cell phone: +251-924850479

E-mail: [Beet964@gmail.com](mailto:Beet964@gmail.com)

**Declaration of informed voluntary consent form for the participant**

I have got full information about the study, the data collector told me that there will be no harm that will occur on me by giving the information for this questioner and the information will not be disclosed for anyone except for the principal investigator and also I know that I will not get any financial support.

I confirmed that the questioner doesn’t have anything that point out/disclose my identity. Because of all this reason I decided to give information and I show my agreement with my signature.

-------------------------------------------- -----------------------------------------

Participant signature Data collector’s signature

______________________

Supervisor’s signature

**Appendix B: Questionnaire for participant interview**

This is the questionnaire to assess “Nurses’ Pain Management Practices for Admitted Patients at the Comprehensive Specialized Hospitals and its Associated Factors 2022”

01. Questionnaire code____________

02. Keble: ____________________

03. Name of Data Collector: ____________ Signature__________ Date: _______

04. Name of Supervisor: ___________ ______ Signature_______ Date: _______

**Part-1: Socio-demographic factors of nurses to nurses’ pain management practices**

| S.NO | Questions | Response |
| --- | --- | --- |
| 101 | Age | Age in year------------ |
| 102 | Gender | 1. Male 2. Female |
| 103 | Educational status | 1. Diploma 2. Bachelor’s degree 3. Masters and above |
| 104 | How many years of experience do you have? | Number of years----------- |
| 105 | Where is your working unit currently? | 1. Medical 2. Surgical 3. Orthopedics 4. Oncology 5. Maternity |

**Part-2: Institution-related factors to pain management practice**

Please read the following questions and give appropriate answer

| S.NO | Questions | Responses | Skip |
| --- | --- | --- | --- |
| 201 | Have you taken any training regarding pain assessment within the last two years? | 1. Yes 2. No | If your response is no go-to Qno 203 |
| 202 | If your answer to QNo201 is yes, when did you take the training? | 1. In the last 6 months 2. Within one year 3. Before one year |  |
| 203 | Have you taken training regarding pain management? | 1. Yes 2. No | If your response is no go-to next part |
| 204 | If your answer to QNo203 is yes, when did you take the training? | 1. In the last 6 months 2. Within one year 3. Before one year |  |

**Part-3: Knowledge of nurses toward pain management practice for admitted patients**

The following are questions designed to assess the knowledge of nurses on pain management for admitted patients. Please answer each question by saying **Yes**-for the question you consider **correct** and **No**- for the **incorrect**. Put this symbol “✔” for the answer you would like to choose.

| S.NO | Questions | Responses | |
| --- | --- | --- | --- |
|  |  | Yes | No |
| 301 | The most accurate judge of the intensity of the patient’s pain is the patient |  |  |
| 302 | Currently, pain is regarded as one of the vital signs of the patient |  |  |
| 303 | Pain should be assessed before and after administering anti-pain drugs |  |  |
| 304 | It may often be useful to give a placebo to a patient in pain to assess if he is genuinely in pain. |  |  |
| 305 | The patient should be advised to use non-drug techniques along with pain medication. |  |  |
| 306 | Distraction, for example, by the use of music or relaxation, can decrease the perception of pain |  |  |
| 307 | Side effects of opioids should be observed at least (20-30) minutes after administration. |  |  |
| 308 | Combining analgesics that work by different mechanisms may result in better pain control with fewer side effects than using a single analgesic agent |  |  |
| 309 | During caring for a patient, providing comfort and positioning may help to reduce muscle tension which in turn, can reduce pain |  |  |
| 310 | Appropriate assessment of pain is the priority for effective pain management |  |  |
| 311 | Acetaminophen and non-steroidal anti-inflammatory agents are effective analgesics for severe pain |  |  |
| 312 | After the initial recommended dose of opioid analgesics, subsequent doses should be adjusted according to the individual patient’s response |  |  |

**A checklist to assess patient-related factors to nurses’ pain management practices for admitted patients**

**Part-4: Patients’ socio-demographic factors to nurses pain management practices**

| S.NO | Questions | Responses | Skip |
| --- | --- | --- | --- |
| 401 | Age of the patient | Age in year------------- |  |
| 402 | Sex | 1. Male 2. Female |  |
| 403 | Marital status | 1. Married 2. Single 3. Divorced 4. Widowed |  |
| 404 | Educational status | 1. Cannot read and write 2. Primary education 3. Secondary education 4. Diploma and above |  |
| 405 | Religion | 1. Orthodox 2. Muslim 3. Protestant |  |
| 406 | Occupation | 1. Governmental employee 2. Private employee 3. Housewife 4. Farmer 5. Daily laborer 6. Merchant |  |

**Part-5: patients’ clinical factors to nurses pain management practices**

| 501 | Ward (disease category) | 1. Medical 2. Surgical 3. Orthopedic 4. Oncology 5. Maternity |  |
| --- | --- | --- | --- |
| 502 | Has the patient undergone any painful procedure to treat his illness? | 1. Yes 2. No | If no, go-to QNo 505 |
| 503 | the latest pain score of the patient | --------- |  |
| 504 | What type of anti-pain had he/she received? | Specify---------- |  |
| **Pain assessment (directly from a patient using a numerical pain scale)** | | | |
| 505 | Think of your current pain status and rate it with the number from 0 to 10 by considering 0-as no pain and 10-as the worst possible pain | ---------------- |  |

**Part-6: An observational checklist to determine pain management practices of nurses from chart**

The following are questions designed to assess the pain management practices of nurses for admitted patients. Indicate the responses by saying “YES” for the activity which has been done by the nurse and “No” for the activity which has been missed by the nurse. Put this symbol “✔” for the answer you would like to choose

| S. No | Questions | Responses | |
| --- | --- | --- | --- |
|  |  | Yes | No |
| 601 | Is the pain score sheet attached and incorporated with the patient’s chart? |  |  |
| 602 | Was the pain assessed and recorded using the standard pain assessment tools at least once over the past 4 hours for this patient? |  |  |
| 603 | Was the pain assessment carried out regularly for this patient? |  |  |
| 604 | Was the patient with pain managed with anti-pain medications? |  |  |
| 605 | Was the pain managed based on WHO pain management standards? |  |  |
| 606 | Was the patient with pain managed with non-pharmacological methods? |  |  |
| 607 | Was the patient’s pain managed before and after every procedure? |  |  |
| 608 | Were the side effects of pain medications monitored as per protocol? |  |  |

**Part-7: A checklist to assess institution-related factors to nurses’ pain management practices**

| S.NO | Questions | Responses |
| --- | --- | --- |
| 701 | Currently, what is the nurse-to-patient ratio of this unit? | 1. 1: 6 2. 1 :8 3. Other……… |
| 702 | Working shifts in the inpatient departments of the institution? | 1. Two 2. Three |
| 703 | Availability of a written policy on “pain is 5th vital sign and must be assessed and managed” | 1. Yes 2. No |
| 704 | Are there pain assessment tools in this ward? | 1. Yes 2. No |
| 705 | Is there pain management guideline in the ward? | 1. Yes 2. No |
| 706 | Have assigned Pain-Free Hospital Implementation Focal person or team | 1. Yes 2. No |
